# Evaluation of knowledge and practices on antibiotic use: a cross-sectional study on self-reported adherence to short-term antibiotic utilization among patients visiting level-1 hospitals in Lusaka, Zambia

**DOI:** 10.1093/jacamr/dlae120

**Published:** 2024-07-19

**Authors:** Martin Kampamba, Bubala Hamaambo, Christabel Nang’andu Hikaambo, Boris Mwanza, Andrew Bambala, Mukumbi Mutenda, Jean M Mukumbuta, Steward Mudenda

**Affiliations:** Department of Pharmacy, School of Health Sciences, University of Zambia, P.O Box 50110, Lusaka, Zambia; Department of Pharmacy, School of Pharmacy, Eden University, Lusaka, Zambia; Department of Pharmacy, School of Health Sciences, University of Zambia, P.O Box 50110, Lusaka, Zambia; Department of Pharmacy, School of Pharmacy, Eden University, Lusaka, Zambia; Department of Pharmacy, University Teaching Hospitals, Lusaka, Zambia; Department of Pharmacy, University Teaching Hospitals, Lusaka, Zambia; Department of Pharmacy, Livingstone University Teaching Hospital, Livingstone, Zambia; Department of Pharmacy, School of Health Sciences, University of Zambia, P.O Box 50110, Lusaka, Zambia

## Abstract

**Background:**

Antimicrobial resistance (AMR) is a global public health problem affecting healthcare systems. Short-term antibiotic non-adherence is thought to be one of the factors contributing to antibiotic resistance. This study aimed to evaluate knowledge and practices towards short-term antibiotic use on self-reported adherence among patients visiting level-1 hospitals in Lusaka, Zambia.

**Methods:**

This was a multicentre institutional-based cross-sectional study conducted among 385 adult participants from 11 September to 30 September 2023 using an adopted structured questionnaire. Analysis of the data involved descriptive and inferential statistics, where significance was determined at *P* < 0.05.

**Results:**

Of the 335 participants, 56.7% displayed good knowledge and 77.3% low adherence towards antibiotic use. 54.6% thought that antibiotics were effective for viral infections, and 43.9% correctly recognized the definition of AMR. Being in formal employment (crude OR: 2.5, CI: 1.08–5.78, *P*: 0.032) was significantly associated with a higher likelihood of good knowledge about antibiotics while being divorced (adjusted OR: 2.5, CI: 1.23–6.10, *P*: 0.013) and having good knowledge (adjusted OR: 2.9, CI: 1.73–5.10, *P* = 0.048) were significantly associated with a higher likelihood of adherence to antibiotics. Regarding antibiotic practices, half (50. 0%) of the respondents had utilized antibiotics in the previous year while 58.2% had taken antibiotics for addressing a common cold. Furthermore, 74% reported to have bought antibiotics without a prescription.

**Conclusions:**

This study found that participants attending level-1 hospitals had relatively good knowledge and poor adherence towards antibiotic use. Additionally, the participants demonstrated poor antibiotic use practices in almost all statements related to antibiotic usage.

## Introduction

The ability of a bacterium to withstand and resist exposure to antimicrobial medicines, threatening the effectiveness of successful infection therapy, is known as antibiotic resistance.^1^ Antibiotic resistance is recognized to be a global public health problem.^2,3^ The extent to which a patient takes medication according to medical or health recommendations is known as medication adherence.^4^ In clinical practice, medication adherence is particularly important and medication adherence has long been a problem since it regularly affects how well a patient responds to therapy.^5^ In the USA specifically, poor adherence alone is cited as the cause of roughly 125 000 fatalities annually and is predicted to contribute to 10% of hospitalizations, resulting in a significant healthcare cost burden ranging from $100 billion to $300 billion due to medication wastage.^6^

According to recent estimations, approximately 4.95 million deaths occurred globally in 2019 due to bacterial AMR, with sub-Saharan Africa recording the highest fatality rates per capita.^7^ These elevated rates result from the widespread misuse of antibiotics, lack of knowledge about antibiotics, insufficient infection prevention and control practices in healthcare establishments, the scarcity of accessible, cost-effective and rapid diagnostic tests, and the demand from patients across Africa.^8,9^ To maintain the ongoing efficacy of existing antimicrobials, a comprehensive approach is required, encompassing measures such as reinforcing healthcare systems and surveillance, enhancing hygiene and infection control practices and promoting judicious use of antimicrobials in both medical facilities and community settings.^10^

Inappropriate use of antibiotics in community, primary care and hospital settings can be attributed to a multifaceted interplay of factors.^11,12^ These include the practices of physicians, patients’ attitudes and beliefs, understanding of antibiotic use, self-medication habits and perceptions of patient–doctor interactions, patient expectations and prior experiences with antibiotics.^13,14^ Hence, effective control of antibiotic usage demands a comprehensive approach involving informed and proactive participation from healthcare professionals, pharmacists, health authorities and consumers.^15^ Understanding their knowledge, attitudes and behaviours regarding antibiotic usage and identifying any educational needs are imperative steps in this process.^16^ Moreover, community-based studies on knowledge and attitudes concerning antibiotics are few and those that have been conducted reported poor knowledge and practices.^17^

In Zambia, several studies have been explored regarding knowledge and practices of antibiotic use among health personnel, students and the public visiting community pharmacies.^12,18,19^ However, no published study has currently evaluated short-term adherence to antibiotics as well as knowledge and practices towards short-term antibiotic use among patients visiting level-1 hospitals in Zambia. As such, the study aimed to evaluate the public’s knowledge and practices related to short-term antibiotic usage, as well as their self-reported adherence to antibiotic treatment.

## Materials and methods

### Study design, site and population

This multicentre institution-based cross-sectional study was conducted among adult patients visiting Chelstone, Chipata and Chawama level-1 hospitals. These three hospitals were randomly selected from a total of six level-1 hospitals in Lusaka District. Participants in the study included adult patients who were receiving secondary care services at the three first-level hospitals.

### Sample size calculation and sampling technique

The single population proportion formula was used to calculate the sample size. $N=\frac{Z^{2}P(1-P)}{e^{2}}$, where *N* = sample size, *Z* = (1.96)2 for 95% CI, *P* = prevalence estimates at 0.5 (based on 50% prevalence), and *e* = maximum tolerable error at 5% (i.e. ±0.05) for the prevalence. Therefore, *n* = 385. No particular recruitment strategy was used; participants were chosen at random. Anyone sitting in one of the approved outpatient pharmacy waiting areas on a weekday between 08:00 and 17:00 h had the opportunity to take part in the study. The inclusions in this study were adult patients who were above 18 years old and were on antibiotics or had used antibiotics before. Subjects attending the designated pharmacy waiting rooms were subjected to a structured face-to-face interview by two trained pharmacists who were not involved in patient care.

### Data collection tool

This study used an adopted structured questionnaire that was developed based on the literature review of comparable studies.^20,21^ The questionnaire addressed a wide range of topics about the participants’ socio-demographic characteristics as well as their knowledge of antibiotics and antimicrobial resistance (AMR), antibiotic use and adherence to short-term antibiotics. Ten statements (six true and four false) were given to the participants to measure their level of knowledge. The first two concerned the identification of antibiotics (amoxicillin is an antibiotic, and paracetamol is an antibiotic); the remaining four concerned the use of antibiotics with or without an indication (antibiotics are effective for bacterial infections, antibiotics are effective for viral infections, antibiotics are indicated to reduce any kind of pain and inflammation, and you can stop taking the antibiotic when you start feeling better); the next two concerned the side effects of antibiotics (antibiotics can kill ‘good bacteria’ of the human ecosystem/antibiotics can cause allergic reactions); and the final two concerned the awareness of AMR (AMR is a phenomenon that occurs when a bacterium loses its sensitivity to an antibiotic, and infection prevention measures limit the development of AMR).

To evaluate practices, participants were asked to answer the following eight questions: ‘Have you taken an antibiotic in the past 12 months? Has your son/daughter taken an antibiotic in the past 12 months? Have you ever taken an antibiotic for a fever or the common cold? Do you keep any leftover antibiotics at home in case you need them later? Have you ever used leftover antibiotics without first consulting your doctor? Have you ever purchased antibiotics without a prescription? Have you ever taken an antibiotic after a phone consultation with the GP, without a clinical examination? Have you ever interrupted the course of antibiotics prescribed to you (when you started feeling better)?

To measure adherence, the Morisky Medical Adherence Scale-8 components were modified for an oral antibiotic treatment; as a result, Questions 2 and 5 were appropriately altered. ‘People sometimes miss taking their medications for reasons other than forgetting were there any days in the last 2 weeks that you missed taking your medication?’ was the original question posed in Question 2. It was changed to read as follows: ‘Most medication missed by people happens for other reasons than forgetting to take it. Did you ever go a day without taking an antibiotic? The initial query in Question 5 was, ‘Did you take all of your medications yesterday? ‘Did you take antibiotics on the last day of therapy?’ was the revised question (Table 5).

We also asked the following questions regarding the cause and impact of non-adherence as well as measures to take to eliminate non-adherence of antibiotics: ‘What do you think are the causes of antibiotic non-adherence? What do you think is the impact of non-adherence of antibiotics on effective therapy? What measures can be made to eliminate non-adherence of antibiotics?’. A translation of the questions to the local language was done by the researcher where the questionnaire was unclear.

### Data analysis plan

Following the collection of data, thorough checks and coding procedures were carried out. The compiled data were then entered into Epi Data version 3.1 and subsequently transferred to STATA version 15.1.^22,23^ Descriptive statistics were utilized, using text, tables and graph representations. Both bivariate and multivariable logistic regression analyses were conducted to determine factors associated with knowledge and adherence. Initially, binary logistic regression was employed to examine the relationship between each independent variable and the outcome variables. Independent variables with a *P*-value of <0.20 in the binary logistic regression were considered for inclusion in the multivariate logistic regression. Throughout the analysis, a significance level of 0.05 and a 95% CI were utilized. Variables with a *P*-value of <0.05 were identified as associated factors of the knowledge and adherence towards antibiotic use.

### Measurement

Knowledge, practices and adherence were the study outcomes of interest. We measured the knowledge level using 10 questions. We awarded a score of 1 for each correct answer, resulting in a minimum score of 0 and a maximum score of 10, respectively. We regarded participants with scores below the mean as having poor knowledge about antibiotic use and those with scores equal to or above the mean as having good knowledge. We measured medication adherence by assigning a yes or no response to Questions 1–7 and using a Likert scale to answer Question 8 (never, rarely, sometimes, often and always). As previously described,^20,21^ the question score was formulated as follows: 1 score was awarded to no answers, and a 1 score was assigned to yes answers, except for Question 5, whose score was reversed (0 score was assigned to no responses, and 1 score was assigned to yes responses). For Question 8, we assigned a score of 1 to responses that occurred never and rarely, and a score of 0 to those that occurred sometimes, often and always. The total questionnaire score ranged from 0 to 8, with scores of 8 indicating high adherence, scores of 6–7 indicating medium adherence and scores of <6 indicating low adherence to antibiotic therapy. Adherence was dichotomized to high (score of 8) and low (score < 7) adherence.

### Ethical approval

This study was conducted after approval by the Ethics Committee from Excellence in Research Ethics and Science Converge (ref no 2023-aug-014) and the National Health Research Authority. Clearance and the letter of request to carry out a study at Chelstone level-1 Hospital, Chipata level-1 Hospital and Chawama level-1 Hospital were written. The participants were made aware that taking part in the exercise was completely optional and that leaving at any time without penalty was permitted at any point during the activity. As much as is practical, consent, privacy and secrecy were honoured. Informed consent was obtained from all participants before questionnaires were administered.

## Results

### Socio-demographic characteristics of the study participants

In this study, 385 questionnaires were distributed to the participants but only 335 responded giving a response rate of 87.2%. The majority 168 (50.2%) of the participants were aged between 18 and 30 years old. Most 196 (58.5) of the participants were females while the majority 146 (43.6%) attained secondary education. Age (= 0.025), education level (0.011) and occupation (0.0001) were significantly associated with knowledge level of antibiotic use (Table 1).

**Table 1.** Socio-demographic characteristics of the study participants according to the knowledge level of antibiotic use (*n* = 385)

Variable	Category	*N* (%)	Poor knowledge	Good knowledge	*P*-value
Age	18–30	168 (50.2)	66 (39.3)	102 (60.7)	0.025
	31–50	117 (34.9)	62 (53.0)	55(47.0)	
	51–80	50 (14.9)	17 (34.0)	33 (66.0)	
Gender	Female	196 (58.5)	83 (42.4)	62 (44.6)	0.681
	Male	139 (41.5)	62 (44.6)	77 (55.4)	
Marital status	Single	167 (49.9)	68 (40.7)	99 (59.3)	0.362
	Married	104 (31.0)	51 (49.0)	53 (51.0)	
	Divorced	64 (19.1)	26 (40.6)	38 (59.4)	
Religion	Christians	324 (96.7)	139 (42.9)	185 (57.1)	0.443
	Muslim	11 (3.3)	6 (54.6)	5 (45.4)	
Education level	None	42 (12.5)	16 (38.1)	26 (61.9)	0.011
	Primary	47 (14.0)	16 (34.0)	31 (66.0)	
	Secondary	146 (43.6)	56 (38.4)	90 (61.6)	
	Tertiary	100 (29.9)	57 (57.0)	43 (43.0)	
Occupation	Formal employment	43 (12.8)	28 (65.1)	15 (34.9)	0.0001
	Informal employment	153 (45.7)	72 (47.1)	81 (52.9)	
	Not employed	139 (41.5)	45 (32.4)	94 (67.6)	

### Participants’ knowledge about antibiotics

When participants were asked about their knowledge of antibiotics, most participants correctly answered Questions 1–3 and 6. However, most participants answered Questions 7–10 wrongly. Regarding the knowledge about correct use and side effects of antibiotics, 189 (56.4%) reported that antibiotics are indicated to reduce any kind of pain and inflammation (Table 2). The overall knowledge level of antibiotic use was scored out of 10 with a mean score of 5.7 (SD ± 1.3) and 190 (56.72%) of the participants displayed good knowledge.

**Table 2.** Knowledge questions about antibiotics were answered correctly by participants (*n* = 335)

Knowledge questions	Answer	Correct answer *N* (%)	Wrong answer *N* (%)
Amoxicillin is an antibiotic	True	242 (72.2)	93 (27.8)
Paracetamol is an antibiotic	False	235 (70.2)	100 (29.9)
Antibiotics are effective for bacterial infections	True	221 (66.0)	114 (34.0)
Antibiotics are effective for viral infections	False	152 (45.4)	183 (54.6)
Antibiotics are indicated to reduce any kind of pain and inflammation	False	146 (43.6)	189 (56.4)
You can stop taking the antibiotic when you start feeling better	False	194 (57.9)	141 (42.1)
Antibiotics can kill ‘good bacteria’ of the human ecosystem	True	84 (25.1)	251 (74.9)
Antibiotics can cause allergic reactions	True	136 (40.6)	199 (59.4)
AMR is a phenomenon that takes place when a bacterium loses its sensitivity to an antibiotic	True	147 (43.9)	188 (56.1)
Infection prevention measures limit the development of AMR	True	122 (36.4)	213 (63.6)

### Unadjusted and adjusted logistic regression of factors associated with knowledge about antibiotics

To determine the factors associated with knowledge levels towards antibiotics, variables with a *P*-value of <0.2 in the unadjusted model were selected to be fitted into the adjusted logistic regression model. In the adjusted logistic regression model, formal employment (crude odds ratio: 2.5, CI: 1.08–5.78, *P*: 0.032) was significantly associated with a higher likelihood of good knowledge about antibiotics (Table 3).

**Table 3.** Unadjusted and adjusted logistic regression of factors associated with knowledge about antibiotics (*n* = 335)

Variable	Category	COR	95% CI	*P*-value	AOR	95% CI	*P*-value
Age	18–30	1					
	31–50	0.6	0.36–0.92	0.023^a^	0.7	0.40–1.34	0.310
	51–80	1.3	0.65–2.43	0.500	1.1	0.45–2.70	0.829
Gender	Female	1					
	Male	1.1	0.71–1.70	0.681	—	—	—
Marital status	Single	1					
	Married	1.4	0.86–2.30	0.180^a^	0.9	0.50–1.66	0.766
	Divorced	1.4	0.75–2.64	0.289	0.9	0.43–1.90	0.790
Religion	Christians	1					
	Muslim	0.6	0.19–2.09	0.447	—	—	—
Education level	None	1					
	Primary	0.5	0.22–0.97	0.042^a^	0.6	0.26–1.48	0.286
	Secondary	1.0	0.49–2.00	0.976	1.1	0.47–2.49	0.849
	Tertiary	1.2	0.50–2.84	0.691	1.2	0.50–1.70	0.640
Occupation	Not employed	1					
	Informal employment	2.1	1.03–4.24	0.039^a^	1.6	0.73–3.47	0.234
	Formal employment	3.9	1.90–8.01	0.0001^a^	2.5	1.08–5.78	0.032^b^

AOR, adjusted odds ratio; CI, confidence interval; COR, crude odds ratio.

^a^ *P* < 0.2 in the unadjusted model.

^b^ *P* < 0.05 in the adjusted model.

### Practices about antibiotic use and self-medication

Table 4 displays reported practices about antibiotic use and self-medication. Of all the participants who were interviewed, about half 168 (50.2%) had used antibiotics in the previous 12 months. When asked about adherence to the prescribed antibiotic regimen, 246 (71.6%) of the study population reported that they had interrupted antibiotic use before they completed the course. The main reasons for stopping taking antibiotics were symptom relief (66.9%), having forgotten to complete the full course of antimicrobial therapy [245, (73.1%)] (Table 4).

**Table 4.** Practices about antibiotic use and self-medication (*n* = 335)

Questions about practice	Yes		No	
	*N*	%	*N*	%
Have you taken an antibiotic in the previous 12 months?	168	50.2	167	49.9
Has your son/daughter taken an antibiotic in the previous 12 months?	132	39.4	203	60.6
Have you ever taken an antibiotic for the common cold?	195	58.2	140	41.8
Have you ever taken an antibiotic for a fever?	101	30.2	234	69.9
Do you keep leftover antibiotics at home because they might be useful in the future?	247	73.7	88	26.3
Have you ever used leftover antibiotics without consulting your GP?	236	70.5	99	29.6
Have you ever bought antibiotics without a medical prescription?	248	74.0	87	26.0
Have you ever taken an antibiotic after a phone consultation with the GP, without a clinical examination?	213	63.6	122	36.4
Have you ever interrupted the course of antibiotics prescribed to you [when you started feeling better]?	240	71.6	95	28.4

### Participants’ level of adherence to antibiotic therapy

**Figure 1.**
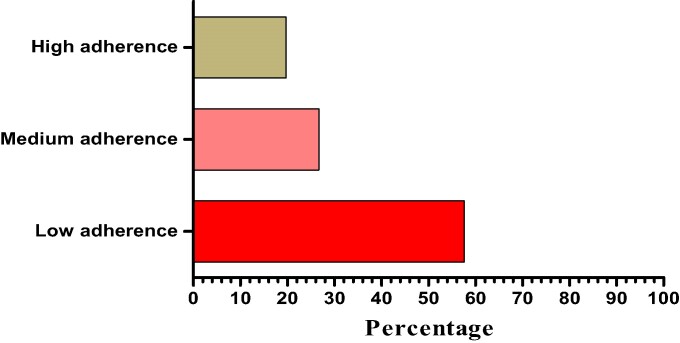
Participant’s adherence to antibiotics therapy (*n* = 335).

Regarding the overall level of adherence to the antibiotic treatment, the majority [193 (57.6%)] displayed low adherence, followed by 76 (26.7%) who exhibited high adherence levels to short-term antibiotic use, and 66 (19.7%) of participants showed medium levels of adherence. Adherence was further dichotomized into low [259 (77.3)] and high [76 (26.75)] (Figure 1). When participants were assessed on adherence to antibiotics, 245 (73.1%) had either sometimes forgotten to take antibiotics or 224 (66.9%) had difficulty remembering to take their antibiotics (Table 5).

**Table 5.** Participants’ level of adherence to antibiotics therapy (*n* = 335)

Questions on adherence	YES		NO	
	*N*	%	*N*	%
Have you sometimes forgotten to take antibiotics?	245	73.1	90	26.9
Most medication missed by people happens for reasons than forgetting to take it. Did you ever go a day without taking an antibiotic?	224	66.9	111	33.1
Have you ever decreased your doses or stopped taking your antibiotics?	218	65.1	117	34.9
When you travel or leave home, have you sometimes forgotten to take antibiotics with you?	205	61.2	130	38.8
Did you take antibiotics on the last day of therapy?	123	36.7	212	63.3
When you felt better, did you sometimes stop taking the antibiotics?	224	66.9	111	33.1
For some people taking medication is a real hassle. Has having to take antibiotics in fixed doses and at the right time ever bothered you?	214	63.9	121	36.1
How often did you have difficulty remembering to take antibiotics?	224	66.9	111	33.1

### Unadjusted and adjusted logistic regression of factors associated with adherence to antibiotics treatment

To determine the factors associated with knowledge levels towards antibiotics, variables with a *P*-value of <0.2 in the unadjusted logistic regression model were selected to be fitted into the adjusted logistic regression model. In the adjusted logistic regression model, being divorced (adjusted OR: 2.5, CI: 1.23–6.10, *P*: 0.013) and having good knowledge (adjusted OR: 2.9, CI: 1.73–5.10, *P* = 0.048) were significantly associated with a higher likelihood of adherence to antibiotics therapy (Table 6).

**Table 6.** Unadjusted and adjusted logistic regression of factors associated with adherence to antibiotic therapy

Variable	Category	COR	95% CI	*P*-value	AOR	95% CI	*P*-value
Age	18–30	1					
	31–50	1.3	0.74–2.28	0.363	—	—	—
	51–80	1.4	0.66–2.29	0.386	—	—	—
Gender	Female	1					
	Male	0.8	0.50–1.41	0.514	—	—	—
Marital Status	Single	1					
	Married	1.4	0.75–2.63	0.283	1.9	0.98–3.78	0.059
	Divorced	2.2	1.04–4.52	0.038^a^	2.5	1.23–6.10	0.013^b^
Religion	Christians	1					
	Muslim	0.6	0.19–2.09	0.447	3.1	0.88–11.1	0.078
Education Level	None	1					
	Primary	0.4	0.13–0.98	0.045^a^	0.4	0.13–1.12	0.080
	Secondary	0.5	0.21–0.97	0.043^a^	0.5	0.21–1.19	0.120
	Tertiary	0.8	0.36–1.69	0.526	0.9	0.40–2.23	0.891
Occupation	Not employed	1					
	Informal employment	0.7	0.34–1.56	0.422	—	—	—
	Formal employment	0.5	0.24–1.16	0.111^a^	—	—	—
Knowledge	Poor	1					
	Good	2.7	1.34–4.96	0.034^a^	2.9	1.73–5.10	0.048^b^

AOR, adjusted odds ratio; CI, confidence interval; COR, crude odds ratio.

^a^ *P* < 0.2 in the unadjusted model.

^b^ *P* < 0.05 in the adjusted model.

### Participants’ perception regarding the cause of non-adherence

**Figure 2.**
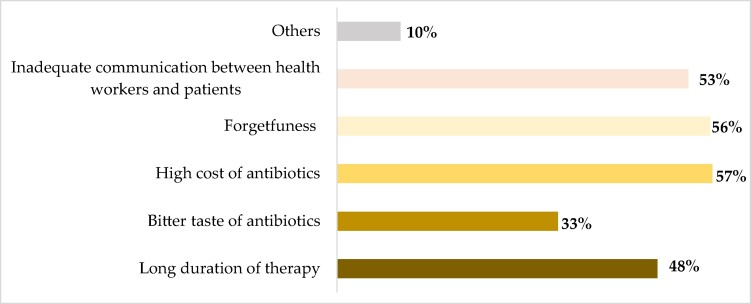
Participants’ perception regarding the cause of non-adherence (*n* = 335).

Figure 2 represents the participants’ thoughts on the reasons for non-adherence to the use of antibiotics. The most common reason for non-adherence to antibiotics was the high cost of antibiotics at 57% seconded by forgetfulness (56%). Other reasons were inadequate communication between health workers and patients and long duration of therapy at 53% and 48%, respectively.

### Impact of non-adherence on the effectiveness of therapy

**Figure 3.**
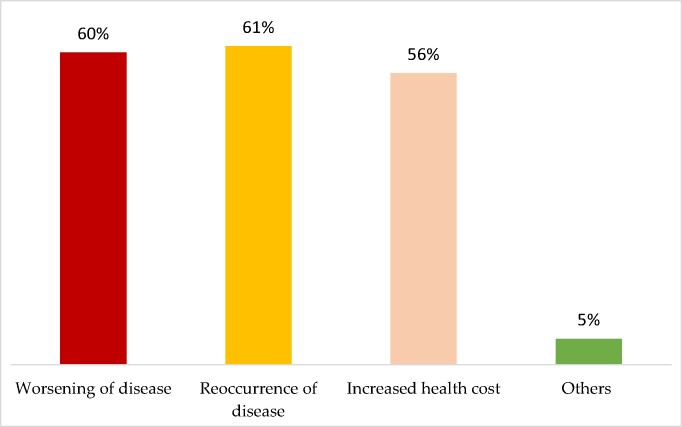
Participant’s responses on the impact of non-adherence on the effectiveness of therapy (*n* = 335).

Figure 3 represents the participants’ thoughts on the impact of non-adherence to the use of antibiotics. Participants were quite knowledgeable as more than half (61%) said one of the impacts was the reoccurrence of the disease, (60%) said worsening of the disease and (56%) said increased health cost.

### Measures to eliminate non-adherence to antibiotics

**Figure 4.**
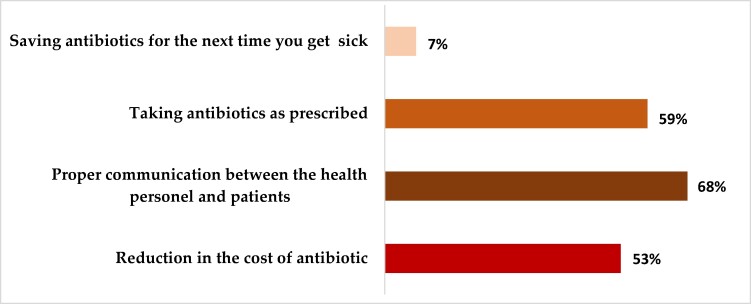
Measures to eliminate non-adherence to antibiotics (*n* = 335).

When assessed on the measures to eliminate non-adherence, the most given measure was proper communication between the health personnel and patients (68%), seconded by taking antibiotics as prescribed (59%). The participants added that a reduction in the cost of antibiotics (53%) would eliminate non-adherence (Figure 4).

## Discussion

Antimicrobials are widely used in the treatment and prevention of infections.^24^ Yet, overuse and improper usage of antibiotics significantly contribute to bacterial resistance, elevating the likelihood of complications and mortality. This results in more challenging management of infectious diseases, higher chances of treatment failure and subsequent increase in healthcare costs.^25^ This study aimed to assess knowledge and practice on antibiotic use as well as self-reported adherence to antibiotic intake.

In the current study, 56.7% of the participants displayed good knowledge about antibiotic use across all knowledge statements. This percentage was lower than that reported in Cambodia (86.5%) and Bangladesh (73.2%)^26,27^ but higher than the percentage reported in Italy (17%), Ethiopia (48%) and Tanzania (20.4%).^21,28,29^ Regarding specific knowledge statements, participants who knew antibiotics play a role in the treatment of viral infection and pain/inflammation represented 54.6% and 56.4%, respectively. Similarly, studies conducted globally concerning the public’s understanding of antibiotic usage indicated a widespread lack of awareness regarding the ineffectiveness of antibiotics in treating viral infections and pain/inflammation.^21,30,31^ In this study, ‘you can stop taking antibiotics when you start feeling better’ was among the knowledge statements where more than half of participants also displayed poor knowledge of antibiotic use. This is in line with a study in Indonesia where half of the participants had contemplated quitting their antibiotics as soon as their symptoms subsided.^32^ The findings of this study are in contrast to what was reported in a study in Italy where it was reported that less than half (30%) interrupted the course of antibiotics when felt better.^21^ This disparity could be due to cultural beliefs and practices that can influence healthcare behaviours. In some African communities, there may be traditional healing practices or cultural beliefs that affect perceptions of antibiotic use, leading to differences in knowledge compared with regions where Western medicine is more widely accepted.

There was also poor knowledge among study participants in this study regarding the AMR phenomena. This aligns with what was reported in Italy where knowledge statements regarding AMR exhibited the uppermost proportion of failure.^21^ These results demonstrated that there is still a global audience that is not fully aware of the existence of AMR and the preventative steps that can be taken to curb it. In the current study, being in formal employment was significantly associated with a higher likelihood of good knowledge about antibiotics. This is in agreement with a study in Indonesia, which found employment status to be associated with antibiotic knowledge. Formal employment likely fosters access to structured educational opportunities and consistent health information dissemination, enhancing individuals’ understanding of antibiotics. Higher education level, occupation and female gender were associated with knowledge about antibiotic use in other studies.^21,33,34^

Regarding antibiotic practices in the current study, 71.6% of participants reported interrupting treatment when they felt better. This is much higher than what was reported in South Africa (48%), Zambia (33.9%), Italy (30.5%) and Ethiopia (52.2%).^21,35–37^ Additionally, 74% of the study participants in the present study reported obtaining antibiotics without a prescription. This is similar to what was reported in one study in Zambia^37^ but substantially higher than what was reported in the previous studies.^38,39^ Stricter policies around over-the-counter antibiotic access could help reduce self-medication.

The findings of our study indicate that a significant proportion, 77.3% of participants, exhibited low adherence levels to antibiotic treatment. This prevalence of low adherence contrasts sharply with previous studies in China, Ethiopia and Jordan, where adherence rates were reported at 13%, 60.1% and 32.1%, respectively.^40–42^ Possible reasons for the disparities could include differences in healthcare systems, cultural attitudes towards medication adherence and variations in study methodologies. In the current study, the low level of adherence reported was mainly due to forgetting (73.1%), discontinuing treatment early when feeling better (66.9%) and the high cost of antibiotics 56.7%. Interventions like reminders, reduced treatment duration when possible and affordable pricing could improve adherence.

In our study, being divorced and having good knowledge about antibiotic use were significantly associated with a higher likelihood of adhering to antibiotic therapy. Improved medication adherence post-divorce can be attributed to a renewed focus on personal health, reduced stress from marital conflict, new support systems and an overall reassessment of priorities. Previous studies also showed that good knowledge had a significant association with adherence to short-term antibiotic use.^41,43,44^ Contrary to our findings, one study found that good knowledge was not associated with adherence to short-term antibiotic use.^45^ Therefore, to enhance patients’ adherence to antibiotic usage in the hospital setting, intervention is vital to address patients’ knowledge of antibiotics.^34^ Contrary to our findings, no published study has found marital status to be significantly associated with adherence to short-term antibiotic use. Some studies associate adherence with factors such as gender, employment, age and seeking information about the prescribed antimicrobial medication.^20,46^

In our study, the majority of participants attributed their non-adherence to short-term antibiotic use to the high cost of antibiotics (57%), forgetfulness (56%) and inadequate communication between health workers and patients (53%). Similarly, other studies found that the high cost of antibiotics and forgetfulness were reasons for non-adherence to short-term antibiotic use.^47,48^

When assessing the measures to eliminate non-adherence to short-term antibiotic use, the most common measure was proper communication between health personnel and patients (68%). One of the independent factors associated with non-adherence was found to be satisfaction with the information provided by the physician.^48^ Therefore, there is a need to enhance communication between patients and healthcare providers, as studies have revealed that health providers often fail to effectively communicate basic treatment plan information to their patients.^49^ Taking antibiotics as prescribed (59%) and reducing the cost of antibiotics (53%), respectively, were the second and third most cited measures to eliminate non-adherence to short-term antibiotic use. Studies have shown that financial barriers are one of the drivers of non-adherence,^50^ and advising patients to set up an alarm to serve as a reminder to take their antibiotics as prescribed can reduce non-adherence to short-term antibiotic use.^51^

In this setting, our findings reveal significant gaps in adherence and practice around short-term antibiotic use. We need multi-pronged interventions through public health campaigns, provider training, adherence support tools and policies to reduce inappropriate antibiotic access. With improved knowledge and appropriate use, we can mitigate antibiotic resistance. This concluding paragraph summarizes the overall findings and connects them to the need for interventions to improve appropriate antibiotic use and awareness. It emphasizes the study’s relevance in addressing the public health problem of antibiotic resistance.

### Study limitations and strength

One of the limitations is that a cross-sectional study is prone to recall bias. This study’s strength was that it was multicentred, which may have provided deeper insights into knowledge, practice and self-reported adherence to antibiotic use among patients visiting first-level hospitals in Lusaka, Zambia.

### Conclusion

In the present study, widespread use of antibiotics was reported, most of it being accessed without prescriptions. The majority of respondents exhibited moderately good knowledge, poor practices and low adherence towards short-term antibiotic use. Therefore, there is a need to consider multifaceted interventions to tackle the determinants of antibiotic misuse at the healthcare system as well as at the patient level.
